# Report of Part of My Surgical Work for the Six Months Ending April 15th, with Remarks*Read at the late meeting of the Georgia Medical Association, Savannah, April 19, 1895.

**Published:** 1895-06

**Authors:** J. B. S. Holmes

**Affiliations:** Atlanta, Ga.


### REPORT OF PART OF MY SURGICAL WORK FOR THE SIX MONTHS ENDING APRIL 15TH, WITH REMARKS.*

BY J. B. S. HOLMES, M.D.,

ATLANTA, GA.

(*Concluded from page* 139.)

CASE 17.—Mrs. S., aged thirty-five, multipara, Georgia. Monday afternoon, February 11th, while walking across her sitting room was suddenly seized with an intense pain in the left iliac region. The pain was so severe that she had to be lifted to bed, in a swooning condition. Her husband, who is a physician, was not

*Read at the late meeting of the Georgia Medical Association, Savannah, April 19, 1895.

at home at the time, but arrived shortly afterwards to find his wife in a fainting condition and suffering most intense agony. He immediately gave restoratives and anodynes and invited two professional friends to see her. She continued to suffer during the night, and notwithstanding the free administration of stimulants she grew weaker and her condition alarming. On the following day there was no improvement, except the suffering was not so acute; pulse rapid, expression anxious, and some distention of the abdomen. On the following morning there was still more prostration, with considerable distention of the abdomen and tenderness; pulse extremely weak and rapid; a well defined peritonitis. Additional counsel was called and a diagnosis of peritonitis concurred in. There was still no improvement in her case, and I was summoned to her home in South Georgia, Wednesday night, the 13th of February. I arrived at one P.M. on Thursday. Found the patient with temperature 102 and pulse 135, extremely pallid, expression anxious, very great distention of abdomen, respiration hurried and embarrassed. The following history was given me: Had been in good health and menstruated regularly; the last regular period occurring eleven weeks before I saw her. Four weeks subsequent to that period the flow came on rather scant and lasted only a day or two. At the regular time it again returned, three weeks before I saw her. This time not so free and lasting only one day. She had suffered some with morning sickness, but not enough to make her think that she was pregnant. In passing my finger into the vagina, I detected a large boggy mass, filling up the left side of the pelvis; the uterus considerably enlarged and pushed far over to the right. I immediately diagnosed tubular pregnancy, with rupture, and urged immediate operation. The husband agreed with me, but the attending physician differed, and opposed surgical procedure. The patient herself, after having everything explained to her, also declined operation. I could not leave her home before one o'clock the following morning. Before the hour of my departure, as she had continued to grow weaker, the husband came to me and said that he felt it his duty to urge an operation upon his wife. He did so and she consented. Strychnia had been given her, hypodermically, freely from the time I saw her, and every preparation was made for operation in the early morning. Her bowels, in the meantime, had been thoroughly evacuated by copious enemata. Early on the morning of the 15th of February, four days after the rupture, in anything but a promising condition, she was anesthetized and operated upon. Upon opening the abdomen the entire cavity was found filled with an enormous quantity of coagulated blood. Enough was removed to find the ruptured and bleeding tube. This was promptly ligated and removed. The coagulated blood (a large wash basin full, I presume, but did not stop to measure it as her condition was so extreme) was removed, the abdomen thoroughly irrigated with boiled water at a temperature of 105, and glass drainage used; the tube remaining in twenty-five hours. I regret not being able to find the fetus which had escaped from the tube into the abdomen, and there doubtless macerated. The operation was done so hurriedly that the clotted blood, when removed, went from the abdomen to the slop basin, which was emptied before I had an opportunity to examine it. She was put to bed in almost a moribund condition. Strychnia and whisky were freely used and hot saline solution thrown into the bowels. I should have used a saline infusion in the veins, but had no instrument with me for doing so. She rallied in a few hours and her recovery was satisfactory in every particular. I had the pleasure of dining with her on the 4th instant. She is entirely well and able to go where she pleases, but of course still weak and anemic.

CASE 18.—Mrs. L., aged thirty-seven, primipara, Georgia. Confirmed invalid since birth of child seven years ago. Suffered greatly from indigestion, no appetite, constipation, headache, extreme nervousness, intense backache, pain in lower abdomen, greater on left side; bearing down sensation in pelvis when standing or walking; very weak. Menstruation regular as to time, but too profuse, lasting from fifteen to eighteen days. She is of phthisical family, her mother and two sisters having died with it. She has a well defined tubercular deposit in the apex of right lung. Examination found left ovary greatly enlarged, prolapsed and lying under the uterus, firmly fixed by inflammatory adhesions. Right ovary also enlarged and extremely tender; perineum lacerated; uterus enlarged and retroflexed. Her ovaries and tubes were removed and uterus elevated February 21st, and she left the sanatorium April 6th, greatly improved in every respect. I would not have operated in this case except to relieve the profuse menorrhagia and the intense pain that she was so constantly suffering. I hoped, by arresting the menstrual flow, to save her strength, and by relieving the pain caused by the imprisoned ovary, to build up her nervous system and improve her digestion, thereby enabling her to be better prepared to combat the pulmonary trouble. Dr. Hardon examined this case with me, and concurred in my opinion in every respect.

CASE 19.—Mrs. DeJ., aged thirty-five, multipara, Georgia. Insomnia, no appetite, poor digestion, constipation, irregular and painful menstruation, which had been profuse for the past few months. Intense backache, pain in left side extending down the thigh, increased by standing or walking. Weak and anemic. Upon examination found the right ovary about the size of a guinea's egg, filled with cysts; left prolapsed, resting on Douglas's cul-de-sac, extremely painful and somewhat enlarged; severe laceration in pelvic floor. Urinary analysis showed a trace of albumin. Both ovaries and tubes were removed February 28th, and a perineorraphy done at the same time. The operation was an easy one and she was put to bed in good condition. For the first thirty-six hours she did well. At the end of that time great irritability of the stomach began; she also complained of headache. Up to this time her kidneys had acted reasonably well. Her bowels were freely emptied by high enema and she was given one-half grain calomel triturates every half hour until six grains had been taken. After giving calomel, several doses of epsom salts were given, but she doubtless vomited most of it. Enough, however, was retained to purge her well. On the third day there was some diminution in the urinary secretion, and digitalin, in doses of one one-hundredth of a grain was given hypodermically every three hours. She became intensely restless and complained of severe headache on the afternoon of the third day, and bromide of sodium in doses of thirty grains was given by the bowel every three hours. Hot applications were made over the loins. At midnight of the third day the nurse noticed some delirium and called me. I found her suffering with the most intense headache. Mustard was applied freely to the spine and bromide of sodium continued. From this condition she soon went into a profound coma. The next morning I had Drs. Todd and Hagan see her with me. Both agreed with me that she was suffering with uremic coma. Sick stomach had very much subsided and she was freely purged with cream of tartar and given pilocarpine hypodermically. This line of treatment with stimulants was continued. She was made to sweat freely with the pilocarpine and sustained by nutritive enema of beef juice, and whisky and lime water and sweet milk given by the stomach. No peritonitis. At no time was she tympanitic nor did she have any tenderness over abdomen; perfect union in both abdominal and perineal incisions, without a drop of pus in either. The above line of treatment was persevered in, but despite all our efforts she died at 10 P. M. Tuesday, five and a half days after the operation. A specimen of the urine was analyzed by Professor Harris, and I quote his report in full: "I have examined the specimen of urine sent to-day, with the following result: Reaction acid; specific gravity 1015; no sugar and but a moderate amount of albumin. The total quantity passed in the twenty-four hours was, I am informed, twenty-seven ounces. On this basis I have estimated that 13.65 grammes of urea were excreted in twenty-four hours—a quantity less than one-half the amount that should have passed. The small quantity of urine—about one-half of what should have been excreted—and the low specific gravity, very pointedly confirm the results which were obtained from the examination for albumin and urea. A considerable number of waxy tube casts were found on microscopic examination. Crystalline deposits were almost entirely absent, doubtless due to the diseased condition of the kidneys. There was neither blood nor pus present." The specimen sent him was drawn thirty-six hours before her death.

CASE 20.—Mrs. P., aged thirty-eight, primipara, Georgia. Severe headache, intensely nervous and hysterical; suffering for fifteen years, since the birth of her child, with pain in lower part of abdomen; horrible backache, greatly increased by walking or standing. No appetite, poor digestion, constipation, frequent urination, but without pain; quite weak and unable to exert herself in any particular. Upon examination I found the uterus retroflexed, the left ovary and tube in Douglas's cul-de-sac, firmly fixed and very sensitive. Right ovary prolapsed and as large as a guinea's egg. Ovaries and tubes removed March 2d, and uterus elevated by shortening the ligaments. Her convalescence was uninterrupted. She left the sanatorium April 7th in a very satisfactory condition in every particular.

CASE 21.—Miss R., aged thirty-three, Alabama. Began to menstruate at fourteen; has always been regular and never suffered a great deal of pain; appetite variable; digestion poor. Had suffered with intense backache for several years, pain extending down the limbs, and greatly increased by walking; very nervous and unable to undergo any exertion. Upon examination found the right ovary prolapsed and nearly as large as a hen's egg. Left ovary firmly fixed in Douglas's sac, and extremely sensitive to the touch. Uterus in good position and normal in size. March 2d, ovaries and tubes removed. The right ovary was found entirely destroyed by cystic degeneration. The left also cystic, had about one drachm of blood in it. The left tube entirely occluded. This patient had had local treatment for more than a year before I saw her. She left the sanatorium April 6th, comfortable in every particular.

CASE 22.—Mrs. C., aged thirty-seven, multipara, Georgia. Had been a confirmed invalid since the birth of her third child three years ago; in bed almost the entire time. Appetite poor; bowels constipated; frequent and painful urination, intense backache, greatly increased by standing; has not walked for three years. When she tried to stand, she said she felt as if everything in the abdomen would drop out, and the dragging down produced such acute and intense pain that she was compelled to again resume the recumbent position. Upon examination I found the pelvic floor *terribly* lacerated, the tear extending from the skin almost to the vagino-uterine juncture. Uterus retroflexed; left ovary as large as a guinea's egg and extremely sensitive; that and the corresponding tube in Douglas's cul-de-sac. Right ovary prolapsed, cystic and extremely tender. Ovaries and tubes removed and uterus elevated March 9th, and her convalescence was satisfactory in every particular. On April 9th the laceration in the pelvic floor was repaired.

CASE 23.—Miss H., aged twenty, Georgia. I regret that this patient could not tell the age at which she began to menstruate. She reports, however, that the flow has always come regularly since it began, and always accompanied with pain. About a year ago she noticed some swelling in lower part of abdomen. For the last six months it had increased considerably, and had given her great pain, so much so as to require the frequent use of anodynes, and within the last three months the abdomen has been so large as to make it quite noticeable. She was sent to me for operation upon a supposed ovarian cyst, and from an examination of the abdomen alone a diagnosis of that kind could easily have been made. The fluctuation, however, was quite indistinct, if felt at all. Upon examination, I found a fluctuating point at the base of the bladder about one and a half to two inches below the meatus. My finger passed high into the vagina could just touch the cervix. On March 9th a free incision was made at the point of fluctuation. She must have discharged quite three quarts or more of dark, grumous material, evidently retained menstrual blood. After the cavity was thoroughly emptied I caught the cervix with a vulcellum forcep and drew it down and passed the sound in the cervix, at the same time passing the index finger of my left hand through the incision into the cavity just emptied. I could distinctly feel the point of the sound, with a membrane intervening between it and my finger. I withdrew the sound, passed the uterine dilator through the cervix and perforated the septum. I then passed two rubber drainage-tubes through the cervix and through the opening in the septum and out at the point of the incision in the sack. The sack was thoroughly irrigated with boiled water at a temperature of 105° and well packed with iodoform gauze. The gauze was removed once a day, each time irrigating the cavity and repacking. The drainage tubes were removed on the third day. The irrigation and packing of the cavity was kept up for seven or eight days. The cervical canal was kept well dilated, allowing the drainage to pass off through it. She was relieved and the incision had entirely healed when she returned home March 30th. This was to me a highly interesting case, clearly one of bicornate uterus, with one cervical canal and a septum dividing the two cornu of the uterus.

Undoubtedly this hematometra had existed since she first began to menstruate.

CASE 24.—Mrs. K., aged twenty-seven, nullipara, Georgia. Began to menstruate at sixteen; no pain for a year, since which time has suffered intense pain during menstruation, which had continued usually about three days. Appetite poor, digestion very bad, severe headache, very nervous, bearing down pain in abdomen; worse on right side and greatly increased since her marriage seven years ago; could not walk or stand without great suffering. On examination I found right ovary and tube firmly fixed in Douglas's sac; ovary considerably enlarged and extremely tender. Left ovary quite tender, somewhat enlarged, but in normal position. Ovaries and tubes removed March 12th. She was put to bed rather weak and was given a hypodermic of strychnia one-thirtieth of a grain once in three hours, and at the end of twelve hours it was discontinued, as her circulation was good. She suffered intensely for the first few hours, so much so as to require two hypodermics of morphia, one-quarter grain each, one given ten and the other fourteen hours after the operation. These relieved her entirely, and during the next twenty-four hours her condition was good in every particular. On the morning of the third day (the 14th) she vomited quite freely. As no gas was passed up to this time, she was given a high enema of soap and water, which passed away without bringing any fecal matter or gas. She was given one-half grain tablet of calomel every half hour until twelve doses had been taken, and strychnia given in one-thirtieth grain doses every three hours, without the passage of either fluid or solid contents; indeed there was no peristalsis. I began to suspect intestinal obstruction from either lymph bands, twisted bowels, or union to the ovarian stump. Her vomiting continued to grow worse, and at noon, fifty hours after the operation, she was given ten grains of calomel, which was repeated in three hours; but, as she vomited freely, I dare say but little of it, if any, was retained. She was then given seidlitz powders, which she also failed to retain. During the afternoon and night of the third day she had a number of enemata of soap and water, turpentine, ox-gall, etc. I was thoroughly convinced by night of the intestinal obstruction. Her pulse began to show evidence of failure and intervals between the strychnia were reduced to two hours. Friday morning, four days after the operation, there was some improvement in the circulation, she was resting some, sleeping a little and not vomiting so frequently, still no peristalsis. By noon the pulse had increased in frequency and lessened in volume, and I decided that we had waited as long as it was prudent and safe, that something must be done or death would ensue. Drs. Earnest and Todd, and my assistants, Drs. Grandy and Davis, saw her with me; all concurring as to her condition and the necessity for reopening the abdomen. At 4 P. M. the abdomen was reopened. Perfect union had taken place in every part of the wound. The ileum, at about its middle, was firmly attached to the ovarian stump on the right side. It had looped around about eight inches and attached itself again, producing a complete obstruction by lymph bands around the bowel. The adhesions were loosened, the lymph bands divided, and every portion of the intestinal tract thoroughly examined. The abdomen was freely irrigated with boiled water at a temperature of 105 and glass drainage used, the tube remaining in twenty-four hours. She was put to bed in a very weak and prostrated condition, having a small pulse of 137. By the free use of strychnia, whisky hypodermically, and hot applications to the body, with whisky and salt water in the rectum, she gradually rallied. From that time her convalescence was uninterrupted. As I write to-day (April 11th) she is walking around her room, and expresses herself as feeling better than she has for several years. She will return to her home on the 15th instant. This patient had had local treatment for three years before coming into my hands.

CASE 25.—Mrs. W., aged twenty-five, multipara, Georgia. In good health up to November, 1894, when she aborted at three months. She did well for ten days or two weeks, when was taken with a chill, followed by high temperature, which continued for some days. She was curetted the last of November or early in December by a couple of most competent gentlemen, Drs. Greene and Davis. Her improvement was marked after the curettage, and in two weeks she was up and able to go about her room for a short time. Some time in December she was seized with a chill, followed by high fever and a general peritonitis. She continued ill until I

saw her early in March. At that time she was extremely weak and in a general septic condition. She was placed upon whisky, strychnia, and chlorate of potash, with a nutritive diet and a hot douche ordered daily, with the use of a mild laxative to keep her lower bowel unloaded. She was brought to my sanatorium March 16th, with a pulse of 110, and temperature 99 in the morning and 101 in the afternoon. She was given strychnia every three hours for the succeeding three days. On the 19th both ovaries and tubes were removed. The adhesions were very numerous, dense, and strong. Both ovaries were about the size of a turkey's egg, both tubes nearly as large as bananas, and both ovaries and tubes filled with pus. The tube on the left side was so soft at its uterine end that the ligature cut through it as it would have done through a piece of soft bread. It was necessary to pass a ligature with a needle deep into the cornu of the uterus to secure the vessels. The abdomen was freely irrigated and glass drainage used, the tube remaining in thirty hours. She was extremely weak when put to bed, but with the free use of strychnia hypodermically, whisky and salt water by the rectum, and whisky by the stomach, she rallied and did well for a week, when her temperature began to rise, and I was convinced that pus was forming somewhere. At the end of the second week I noticed slight fluctuation in the track of the drainage tube, and opened the incision at this point, when two ounces of vile smelling pus was discharged. Peroxide of hydrogen was poured into the opening and a dressing of bichloride gauze applied. This was repeated daily. Her temperature is now normal (April 11th), her appetite good, she is sleeping well; a very slight discharge of pus, and her condition in every particular satisfactory. Her recovery is now assured.

CASE 26.—Miss B., aged seventeen, Georgia. Began to menstruate at fifteen; always lasting a week and quite profuse, no pain. Has always suffered with backache; constipation, poor digestion, poor appetite, slept badly and had constant headache. Two years ago she began to have slight nervous attacks, followed by typical epileptic seizures. She has, in the last two years, had from one to three severe paroxysms each month, with from fifteen to twenty-five mild ones each day; a typical case of epilepsy. Her family history is good; no history, as far as I can ascertain, of epileptic trouble in either the mother's or father's family. No specific trouble, no history of convulsions in childhood, or of an injury of any character. She has been treated by a number of competent gentlemen, and has had the bromides freely administered for the past two years, with little or no apparent benefit. Upon examination I found the right ovary prolapsed, tender, enlarged. The left considerably enlarged, exquisitely tender; certainly as much so as any I have ever touched. The gentlest touch gave her the most intense pain; it was resting directly behind the uterus, very low in Douglas's sac. I explained to her and her mother that I could find no other cause for her epileptic trouble, which, in my opinion, was from reflex irritation from the diseased ovaries. At any rate, a very thorough line of treatment had been given without benefit to her, and while I could not promise a cure, I thought the removal of this supposed cause offered her more promise of relief than anything else. Furthermore, I considered the removal of the ovaries in her case as justifiable, as she certainly was not a proper subject to bear children, the effect of heredity being a well known factor in the etiology of epilepsy. After explaining the condition of things to her and her mother, they were both anxious that the operation should be done. Both ovaries and tubes were removed March 23d. Found both ovaries almost entirely destroyed by cystic degeneration, the left entirely so; tubes in fairly good condition. Since that time she has had no severe paroxysms and very few of any character. In the last seventy-two hours she has had only two, both extremely light. I shall watch her case with great interest and confidently expect and believe she will be cured.

CASE 27.—Mrs. M., aged thirty-three, multipara, Georgia. For the past several months had suffered with pain in the pelvis of a sharp lancinating character. Copious leucorrhea, very acrid, and frequently tinged with blood. She had menstruated rather irregularly and always scant, but never with much pain. Upon examination the cervix was found enlarged, eroded, and nodular. Family history shows three near relatives having had cancer. High amputation of the cervix was done April 9th, removing all diseased tissue. The mucous membrane of the cervical canal was stitched to that of the vagina, and the mucous membrane of the anterior and posterior portion of the vaginal wall were brought together on either side of the cervical canal. Before doing this, however, the uterus was thoroughly curetted and irrigated. A short Wyley's plug of hard rubber was passed into the uterus, and the vagina packed with iodoform gauze. It might have been better and safer to have done a hysterectomy in this case, but the body of the uterus was not at all involved, and the patient begged so hard that it be left, that I complied with her wishes, with the understanding that she would submit to it if any return of the disease was noticed.

Case 50.—Mrs. R., aged forty, multipara, Florida. For the past five or six years menstruation has been very profuse; bowels constipated; appetite, poor; suffered great pain in lower abdomen, especially when standing or walking; slight laceration of the cervix; perineum badly torn, involving the sphincter; unable to control contents of bowels since birth of last child six months ago. April 9th, uterus curetted and perineorraphy done. This patient has a very large and tender ovary, which will doubtless, in the near future, require removal.

Case 51.—Mrs. B., aged fifty-two, multipara, Georgia. General health fairly good, menstruation regular. Fifteen months ago noticed a hard lump about the size of a small marble in right mamma. It gradually grew until it was the size of a hen's egg when she consulted me on the 12th instant; slightly tender to the touch, always painful when right arm is used. Had sharp and lancinating pains dart through the mamma and right side of chest. No involvement of axillary glands. No history of cancer in the family. She was prepared, as in case No. 1, and operated on the 13th instant, in a similar manner. As I write (15th instant) the drainage-tube has just been withdrawn; dressings over incision remain undisturbed. Her temperature, up to this time, has only reached 99°, and her condition in every particular is as satisfactory as I could wish.

Ether was administered to nearly all the foregoing patients. Chloroform was used in the second operation upon case No. 24. I believe if the secondary effect of ether upon the kidneys was closely watched we would find as many or more deaths traceable to it than to chloroform, both primarily and secondarily. The risk with ether is undoubtedly greater secondarily than primarily. Many deaths are caused by it that are attributed to other causes. When carefully used, and properly administered, I believe chloroform to be quite as safe, and it is much more satisfactory. The struggling of the patient when being anesthetized is done away with, and the horrible sick stomach that so frequently follows the use of ether rarely occurs with chloroform. The return to consciousness after taking chloroform is rarely accompanied with vomiting or severe nervous manifestations. I am daily more inclined to the use of chloroform, and expect, in the near future, to see the tide of professional opinion turn in its favor. Before administering it, if my patient is at all weak, I always give a hypodermic of one-thirtieth or one-twentieth of a grain of strychnia. I use altogether an Esmarch's inhaler for administering it. There is still a great diversity of opinion among many able surgeons as to the relative safety of the two anesthetics in the presence of kidney disease. Some even claim that chloroform produces more renal trouble than ether. I shall always, until experience proves to the contrary, give chloroform the preference in any diseased condition of the kidney, unless there are other potent reasons why ether should be used. In the fatal case recorded, No. 19, chloroform was used, and I believe had ether been used in this case, death would have occurred within forty-eight hours. I shall never operate again in any case where albumin, in any quantity, is shown in the urine, without a thorough and quantitive test for urea, and a microscopical examination for casts, unless it be one of great emergency. If the quantity of urine is near normal, and the quantity of urea in it not notably deficient, hurried operations may be done even if we have albumin and some casts. If, however, the quantity of urine passed is small and the amount of urea notably deficient, I would only operate for pus accumulations, or very large tumors, producing severe pressure symptoms. Where I have them under control, I like for my patients to flush the kidneys for three or four days preceding an operation with Lithia water, and I always use it for two or three weeks afterwards. It is also well, before surgical procedure, in those cases where you have the preparation of the patient, to see that the skin is clean and in active condition. We are all in the habit, I am sure, of giving too much ether and chloroform. If properly used, after the patient is thoroughly anesthetized, very little is required to keep up complete anesthesia. I find strychnia in my surgical work the most reliable of all heart tonics. Where I have the preparation of the patient, unless she is very strong, I always give one-thirtieth of a grain of strychnia every four to six hours three or four days before the operation; and if she shows evidences of shock after operation, I give it freely; watching for its physiological effects, and continue it for several days until her pulse is sufficiently strong. I consider it of much greater value as a stimulant and heart tonic than brandy. For surgical shock, I have very little faith in digitalis; it has always been disappointing to me. Case No. 17 has impressed me with the importance of always going prepared to use saline infusions, which have, I think, a most valuable place in the treatment of shock from loss of blood.

While I have always deprecated the use of opium, especially in abdominal work, and have used it with great care and caution, an increasing experience convinces me that those cases where it is not given at all do much better, and their convalescence is more satisfactory in every respect. While the pain after abdominal sections is frequently severe, it rarely continues but a short while. Where the pain is so intense and threatens to exhaust the patient from continued suffering, I think it advisable to use it in moderation. I think many of the cases of bowel obstruction, whether due to paresis, lymph bands, or adhesions, are traceable to the use of opium. I am convinced that the adhesions in case No. 24, where the abdomen was reopened, were due to the paralyzed or quiescent condition of the bowels, so rendered by the two doses of morphine given after the operation. While all peristalsis was arrested and the bowel was lying quietly against the ovarian pedicle, the lymph was rapidly pouring out and the constriction produced. Had the peristalsis kept up no adhesion would have occurred. Just here let me enter a plea for the early reopening of the abdomen in all cases of diagnosed or strongly suspected post-operative obstruction. Do not let your patient die without giving her a chance for her life.

In my laparotomy cases, where it has been necessary to elevate the uterus for retroflexion or retroversion, I have done so by shortening the round ligaments, by including them in the ligatures that encircle the ovarian pedicle. In the few cases in which I have done this, the uterus has remained well forward, while it is still allowed free movement. It is not fixed against the bladder, causing great irritability of that organ, that ventro-fixation sometimes does.

There are many other points I would like to discuss, but the length of this paper forbids. I herewith submit for your inspection a few specimens that I have removed from women who have been the victims of one to three years of the so-called conservative treatment with iodine, iodized phenol, nitrate of silver, nitric acid, carbolic acid, and other similar caustics.

---

### LOCALIZED AND PERSISTENT ERYTHEMA FROM ANTIPYRINE.

Dr. L. Brocq, of Paris, has observed four cases of localized and persistent erythema from the use of antipyrine. They occur in either round or oval plaques, occasionally of quite large size, even eight centimeters in diameter. They appear isolated and scattered here and there on the surface of the body, without symmetry and generally few in number. In the beginning the color is reddish gray, and they are often accompanied by burning sensations. There is rarely actual pruritus; later they become indolent. The erythema disappears, little by little, leaving a brown pigmented spot, which after repeated eruptions, which always select the same spot, may become blackish brown. Sometimes small vesicles are formed, and nearly always there is lamellar desquamation on disappearance of the erythema. The plaques are quite distinctly outlined, and are sometimes associated with a distinct swelling of the skin. The pigmentation is the last to disappear; in the smaller plaques it is no longer observed after two or three weeks, if the drug be left off; in the larger ones it persists longer. If the drug be still continued, a new erythematous outbreak appears on the old sites with increase of pigmentation. In some cases the eruption may last from a month to six months.—*Jour. Cut. and Gen. Ur. Dis.*, March, 1895.

